# First molecular detection of the presence of honey bee viruses in insects, *Varroa destructor* mites, and pollinated plants in an isolated region of Armenia

**DOI:** 10.14202/vetworld.2023.1029-1034

**Published:** 2023-05-15

**Authors:** Hranush Arzumanyan, Hranush Avagyan, Henry Voskanyan, Liana Simonyan, Jon Simonyan, Zara Semirjyan, Zaven Karalyan

**Affiliations:** 1 Laboratory of Cell Biology and Virology, Institute of Molecular Biology of NAS RA, Yerevan, Armenia; 2 Experimental Laboratory, Yerevan State Medical University, Yerevan, Armenia; 3 Scientific Center for Risks Assessment and Analysis in Food Safety Area, CJCS, Yerevan, Armenia; 4 Department of Medical Biology, Yerevan State Medical University, Yerevan, Armenia

**Keywords:** *Apis mellifera*, honey bee virus, polymerase chain reaction assay, pollinated plants, *Varroa destructor*

## Abstract

**Background and Aim:**

Recently, viral diseases of honey bees (*Apis mellifera*) have presented an increasing threat to beekeeping. This study aimed to examine the presence of honey bee viruses in *Apis* and non-*Apis* bee species, the mite *Varroa destructor*, and pollinated plants in Armenia.

**Materials and Methods:**

Sampling was performed in Tavush Province, in the northeast of the Republic of Armenia, from August to November 2019. Overall, 200 *A. mellifera* bees, 50 *V. destructor* mites, and 20 wasps were collected (corresponding to three bees, five mites, and 2–11 wasps in each investigated sample) and homogenized for RNA isolation and detection of viruses. Ten pollinated plants were taken from each plant, and 2 g of each sample was used for homogenization. In each investigated case *Apis mellifera, Varroa destructor, Vespula germanica* and plants received percentages of the virus presence.

**Results:**

Six important honey bee viruses (acute bee paralysis virus [ABPV], deformed wing virus [DWV], *A. mellifera* norovirus [ANV], Lake Sinai virus-2 [LSV-2], Big Sioux River virus [BSRV], and *A. mellifera* filamentous virus [AmFV]) were detected in samples by polymerase chain reaction. Our results showed that DWV, ANV, and ABPV were the most common viruses in honey bees. All viruses were detected in wasps, but LSV-2 and ANV were present in almost all samples.

**Conclusion:**

Our results showed that almost all viruses were present in *V. destructor*. Although ANV is very common in honey bees, it did not appear in any mite samples. Our study indicates that viruses typically associated with honey bees were also actively infecting wasps. Our data suggest that the survival of viruses in plants can be an important source of seasonal transmission of viruses to bees. In addition, pollinated plants can potentially serve as reservoirs for honey bee viruses.

## Introduction

Recently, viral diseases of honey bees (*Apis mellifera*) have presented an increasing threat to beekeeping and a wide range of crops, including fruit, vegetables, oilseeds, and legumes that are pollinated by bees. Moreover, viruses infecting honey bees can affect many other insect species, thereby threatening ecosystems more profoundly than previously thought [1–5]. In the past decade, the emergence of new strains of previously unknown viruses has been reported; this development, together with the sending of bee packets, has meant that viruses can easily become established in regions where they were previously absent [6, 7]. Viral transmission can occur horizontally (e.g., via food and/or vectors) or vertically (from mother to offspring). The mite *Varroa destructor* plays an important role in spreading viral diseases by acting as a reservoir and transmitter of honey bee viruses [2, 7–10]. Many studies have elucidated the presence of the virus in other hosts that can be considered potential sources of virus transmission [11, 12].

Some of the viruses cause severe diseases and are distributed worldwide. The prevalence of such viruses has also been reported in neighboring countries for example, in the Islamic Republic of Iran [6, 13]. These viruses include acute bee paralysis virus (ABPV), *A. mellifera* norovirus (ANV), *A. mellifera* filamentous virus (AmFV), deformed wing virus (DWV), Big Sioux River virus (BSRV), and Lake Sinai virus-2 (LSV-2).

This study aimed to examine the presence of honey bee viruses in *Apis* and non-*Apis* bee species and the mite *V. destructor*, in Armenia, to elucidate possible floral resources that can be considered as natural settings where viruses spread.

## Materials and Methods

### Ethical approval

The study was reviewed and approved by the Ethics Committee of the Institute of Molecular Biology NAS RA (IRB 06042021/1, 2021).

### Study period and location

**Figure-1:**
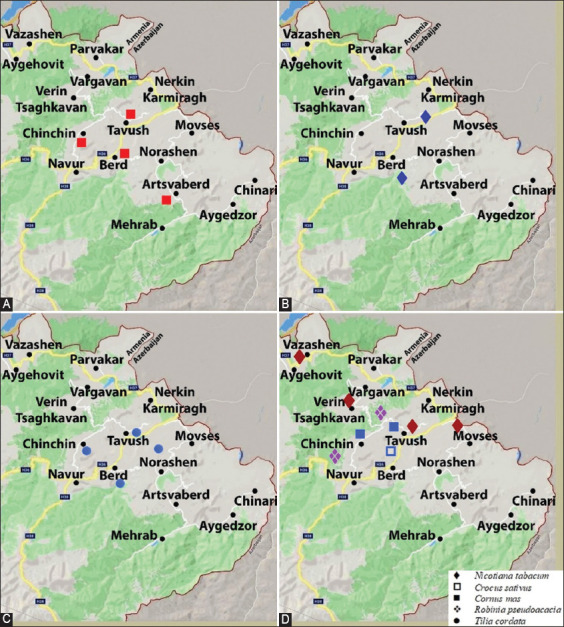
Map of the sampling sites with honey bee virus-positive samples in different arthropods and in pollinated plants. (A) Honey bee samples. (B) *Varroa destructor* samples. (C) *Vespula germanica* samples. (D) Samples were obtained from different pollinated plants [Base map source: https://www.google.com/maps/].

The study was conducted from August to November 2019. Sampling was performed in Tavush Province (Data present in Figures-1A-D) in the northeast of the Republic of Armenia. The geographical area of interest for this study was the north-eastern region of the Republic of Armenia. The Tavush Province is situated in the north-eastern part of the Armenian Republic and the south-east is bordered by the Azerbaijan Republic, having a 300 km border with Azerbaijan (Figure-1, https://www.google.com/maps/)), and is comparatively isolated from the other regions of Armenia. The territory is mainly mountainous and rocky hillsides are covered by forests.

### Viruses

The following viruses were considered in the investigation: ABPV, ANV, AmFV, DWV, BSRV, LSV-2.

### Arthropod and plant sampling

Six aviaries of *A. mellifera* Caucasia, a bee species that is neither endangered nor protected, that are dedicated to honey production were studied. At least 100 adult bees from each apiary were collected. Ten honey bees were used for each sample which was used in viral detection with a polymerase chain reaction (PCR) method.

For mite sampling, five aviaries were investigated for the presence of Varroa mites and 50 samples (5 *V. destructor* were used in each sample) were collected. Wasps (*Vespula germanica*) were sampled on two occasions at 20 samples (two and 11 were per sampling) near the apiaries in the fall of 2019.

The following pollinated plants were used in the investigation: *Crocus sativus*, *Tilia cordata*, *Nicotiana tabacum*, *Cornus mas*, and *Robinia pseudoacacia*. At least ten samples (2 g in weight for each sample) of each experimental plant consisting of entire inflorescences were collected.

All obtained samples were put in a cooler bag and immediately sent to the laboratory, where they were frozen at −26°C. All samples were homogenized in a ceramic mortar with sterile diethylpyrocarbonate-treated water for nucleotide extraction. In each investigated case *Apis mellifera, Varroa destructor, Vespula germanica*, and plants received percentages of the virus presence.

### RNA extraction and polymerase chain reaction (PCR) assay

Homogenized samples were subjected to nucleic acid extraction using a HiGene™ Viral RNA/deoxyribonucleic acid (DNA) Prep Kit (BIOFACT, Yuseong-gu, Darjeon, Republic of Korea), in accordance with the manufacturer’s instructions. RNA/DNA samples were then reverse-transcribed with a REVERTA-L kit (AmpliSens Biotechnologies, InterLabService, Moscow, Russia). Viruses in samples were detected by polymerase chain reaction with a Q1600 Real-time PCR device. The assay used the previously described primers for ABPV, AmFV, DWV, LSV-2, [14], and BSRV [15]. ANV primers used for amplification were designed based on *Apis* norovirus isolate RI-11 sequence (GenBank: KY354240.1) of genes in FASTA format and ordered from Integrated DNA Technology-IDT (https://www.idtdna.com/pages) as follows: All primers are listed in Table-1. The PCR reactions were implemented using a BioMaster HS-Taq PCR kit (Biolabmix Moscow, Russia) following the manufacturer’s protocol. All complementary DNAs were amplified by PCR for the related viral target, and amplicons were visualized on 2% agarose gel and stained with GelRed (Biotium, USA).

**Table-1:** Oligonucleotide primer pairs employed in PCR assays.

Viruses	Primer Sequence (5’-3’)
ABPV	F: TTATGTGTCCAGAGACTGTATCCA R: GCTCCTATTGCTCGGTTTTTCGGT
ANV	TTA: F: TGGGTGAAAACAACAGGGCT R-R: R: ACGTGTCCTGTAGCGTTGAG
AmFV	F: CGCATGTACCAACAACTCGTAC R: CACAGTTGGTGTAGCGCAGT
DWV	F: ATCAGCGCTTAGTGGAGGAA R: TCGACAATTTTCGGACATCA
BSRV	F: GTGCAGCTTTATGCGTTGCC R: CCGCTGTTGAGAATAAGGATATCCAGG
LSV-2	F: CGGCCGGTCTAGCGTGGTTG R: TGGCAAGCTGTGACGAATCCCT

PCR=Polymerase chain reaction, ABPV=Acute bee paralysis virus, ANV=*Apis mellifera* norovirus, AmFV=*Apis mellifera* filamentous virus, DWV=Deformed wing virus, BSRV=Big Sioux River virus, LSV-2=Lake Sinai virus-2

## Results

### Polymerase chain reaction detection of six bee viruses in honey bee (*A. mellifera*), mite (*V. destructor*), and wasps

**Figure-2:**
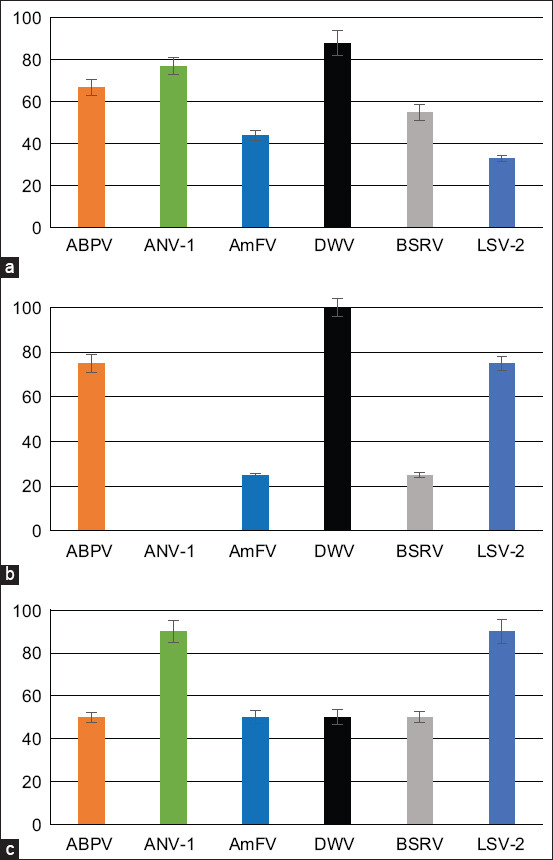
Distribution of honey bee viruses in arthropods Tavush Province. (a) Viruses detected in honey bees. (b) Viruses detected in *Varroa destructor*. (c) Viruses detected in *Vespula germanica*.

The data on the PCR detection of six bee viruses in honey bee and wasp samples are shown in Figure-2. In honey bees, DWV was observed at the highest frequency (almost 90%), followed by ANV and ABPV (about 70%), then BSRV and AmFV (about 50%), and finally LSV-2 (in only 33% of the investigated honey bees) (Figure-2a).

The presence of DWV was detected in all investigated samples of *Varroa* mites. Meanwhile, ABPV and LSV-2 were observed in 70%–80% of investigated apiaries AmFV and BSRV were described as being rare (25%), and no *Varroa* samples had ANV (Figure-2b). All investigated viruses were detected in wasps; however, LSV-2 and ANV were present in almost all samples and all other viruses were present in about 50% of them (Figure-2c).

### Polymerase chain reaction detection of six bee viruses in pollinated plants (*N. tabacum, C. sativus, R. pseudoacacia, C. mas*, and *T. cordata*)

**Figure-3:**
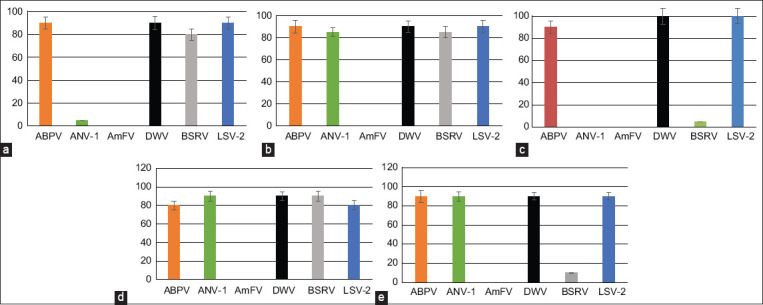
Distribution of honey bee viruses in pollinated plants: (a) Viruses detected in *Nicotiana tabacum*. (b) Viruses detected in *Crocus sativus*. (c) Viruses detected in *Cornus mas*. (d) Viruses detected in *Tilia cordata*. (e) Viruses detected in *Robinia pseudoacacia*.

We investigated the two important pollinated fall plants (*N. tabacum* [Figure-3a], *C. sativus* [Figure-3b]) and three common pollinated trees in spring/summer (*C. mas* [Figure-3c], *T. cordata* [Figure-3d], and *R. pseudoacacia* [Figure-3e]). *Apis mellifera* filamentous virus was absent from all plant samples. All viruses were found at different frequencies in flowers and trees. More often, plant samples were identified as ABPV, DWV, and LSV-2 viruses. *Apis mellifera* norovirus was found in *Crocus*, *R. pseudoacacia*, and *T. cordata*, but was almost absent from *N. tabacum* (about 5%), and was completely absent from *C. mas*. The presence of BSRV was observed in *C. sativus*, *N. tabacum*, and *T. cordata*. However, this virus was uncommon in *C. mas* and *R. pseudoacacia* (5%–10%).

## Discussion

Honey bees are the most important crops and pollinators of wild plants. In terms of their value, these pollination services provide billions of dollars of added value to agriculture [16–20]. Most viral infections pass without clinical manifestations of characteristic disease signs. However, viral infestations are of great concern as they can damage honey bees at various developmental stages, such as egg, larva, pupa, adult worker, drone, and queen [21].

The findings described in this paper demonstrate for the 1^st^ time the infection of honey bees by viruses in Armenia. Among the studied honey bee viruses, DWV and ABPV are well-characterized viruses that cause overt disease and are the most important in beekeeping [22–25].

Based on our results, besides DWV and ABPV, ANV was the most common virus detected by PCR in honey bees. *Apis mellifera* norovirus is an unclassified positive-strand RNA positive-strand virus associated with *Drosophila* species, which was recently detected and identified [7]. There is limited information about the geographical distribution and transmission routes of ANV.

In the past two decades, particular attention has focused on discovering bee viruses in different arthropod species and their possible transmission routes. It is well known that the globally distributed ectoparasite *V. destructor* is a vector for viral pathogens of *A. mellifera*. The presence of honey bee viruses in *V. destructor* has been described in many articles; moreover, *V. destructor* infestation of *A. mellifera* colonies can introduce several important honey bee viruses and/or promote their replication [26–29], so the detection of almost all viruses in our investigations was not unexpected. Interestingly, even though ANV was present in honey bees and wasps, it did not appear in any samples of mites.

Recently, research has been intensifying on *Vespula* wasps as carriers of honey bee pathogens. These wasps may play an important role in virus transmission and be a reservoir for emerging honey bee viruses.

Our study showed that viruses typically associated with honey bees also actively infect German wasps. All viruses were detected in wasps, but LSV-2 and ANV were present in almost all samples. The literature describes that the wasp pathogen fauna is similar to that of honey bees [30, 31], so the similarity of viruses between honey bees and wasps was anticipated.

It is currently considered that floral resources can act as platforms for the spread of pathogens between commingling pollinator species and provide transmission routes through which these pathogens can be acquired. However, despite intensive research on this issue, the role of plant species and floral trait variability in shaping transmission dynamics is almost completely unexplored.

Dogwood (*C. mas*) is one of the earliest flowering plants (starting in early spring), attracting bees and providing them with an important source of nutrition. Meanwhile, *R. pseudoacacia* flowering usually takes place in May to June. Short-term flowering of linden (*T. cordata*) usually occurs in early July. Thus, the flowers of these trees are not pollinated by bees before sampling from several weeks to 3–4 months. However, dried flowers continue to maintain the presence of all viruses, except AmFV.

These findings support the assertion that flowers serve as bridges in the transmission of viruses between bees [12]. According to the data, we can assert that the survival of viruses in plants can be an important source of seasonal transmission of viruses to bees. In addition, pollinated plants can serve as possible reservoirs for honey bee viruses.

## Conclusion

These findings lead us to suppose that the survival of viruses in plants can be an important source of seasonal transmission of viruses to bees. Also, pollinated plants can serve as possible reservoirs for honey bee viruses.

## Authors’ Contributions

HAv and ZK: Conceptualization, methodology, and data curation. HAv and ZS: Resources, funding acquisition. HAv and ZK: Project administration, supervision, writing–review, and editing. HA, HAv, JS, and LS: formal analysis, visualization. HAv, HA, JS, LS, and ZS: Investigation, validation. All authors have read, reviewed, and approved the final manuscript.
